# Supplementary material for: Learning curve and surgical outcome of robotic assisted colorectal surgery with ERAS program
Source: Sci Rep. 2022 Nov 29;12:20566. doi: 10.1038/s41598-022-24665-w (PMC9709162; doi:10.1038/s41598-022-24665-w)
Supplement: Supplementary file 2 — Supplementary Information 2. [file 41598_2022_24665_MOESM2_ESM.zip › RAL Raw data-Ñ[▒K/085/OP N_ñw╝╨░O▒Kñσ.pdf]

W52 / W52

臺中榮民總醫院
手術室護理記錄

[redacted]

基本資料

- 室別： 開刀房-17室，手術科別： CRS，手術類別： 常規，日期： 2021/05/20
- 進入時間： 0735 ； 麻醉開始時間： 0745 ； Time Out時間： 0920 ； 劃刀時間： 0922 ； 手術結束時間： 1455 送出時間： 1500 ；
- 麻醉方式： GE

  診斷： Malignant tumor of rectum SP CCRT

  手術名稱： Robot Assisted Low Anterior Resection + Loop ileostomy

  手術局部用藥： nil

  術前預防性抗生素：
  - 抗生素名稱一： Veterin 1GM IVD由麻醉護理師執行
  - 時間： 0855
  - 上次簽章: 周筱涵

  ，追加預防性抗生素：
  - 抗生素名稱一： Veterin 1GM IVD由麻醉護理師執行
- 手術醫師： 蔣鋒帆，楊曙榮
- 麻醉醫師： VS 齊光彪
-
  - 刷手護士A ： 陳盈妤，時間： 0735，刷手護士B ： 吳婉寧，時間： 0735，接替刷手一： 周筱涵，時間： 1130，接替刷手二： 吳婉寧/周筱涵，時間： 1200
  - 流動護士A ： 周筱涵，時間： 0735，接替流動一： 陳盈妤，時間： 1100，接替流動二： 張秀敏，時間： 1230

皮膚狀況

A-V Shunt： 無

術前： 完整

記錄者： 上次簽章: 周筱涵

術後： 完整

記錄者： 上次簽章: 張秀敏

手術準備

手術臥式 單一手術臥式

-
  - 手術臥式： 截石臥式
  - 使用附件： 墊肩，其它： 海綿墊*3
  - 皮膚準備： 10%Aq Betadine，Hibiscrub，2% Chlorhexidine
  - 電燒： 使用電燒: 電極片位置： 右大腿，記錄者： 上次簽章: 周筱涵

止血帶T： 無

植入物

- 植入物名稱/號碼/數量/部位： coviden GIA 80*4.8/rectum

，記錄者： 上次簽章: 張秀敏

標本送檢

- 細菌培養 無
- 細胞學 無
- 冰凍切片 無
- 病理標本

  組織名稱一： rectum

  ,

  組織名稱二： IMA root

  ,

  組織名稱三： distal cut end

，更多組織名稱
    - 組織名稱四： pelvic wall tumor
- 截肢 無

標本交付 標本： 4 袋，標本： 0 盒，標本： 0 瓶，細胞學： 0 管，Culture Tube： 0 管，被交付醫師： CR 楊曙榮，

- 記錄者： 上次簽章: 張秀敏
- 時間： 1317

傷口術後敷料

傷口敷料： 傷口敷料： 無

- 請於圖上標示部位：

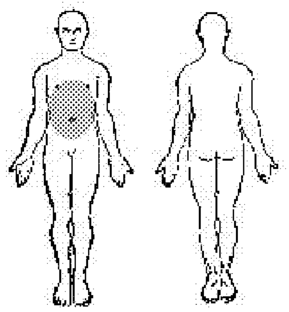

腹部torcar洞

- 傷口敷料： menport

導管

導管	天數	導管資訊	插入日期	插入時間	更換日期	拔除日期	拔除時間	拔除原因	預計拔除/更換	預計拔除/更換日期
Rod,Stoma Supporting (1)	8	部位:迴腸造口;	2021/05/20	1400					拔除	20210524

用物計數

- 手術中出血量： 少 c.c.
- 手術中尿量： 700 c.c.
-
    - 其它： 無

用物計數： 多切口同時縫合

- 1.器械： 正確，2.縫針： 正確，3.刀片： 正確，4.紗布： 正確，5.腹部墊： 未使用，6.紗球： 未使用，7.PEANUTS ： 未使用，8.紗條： 未使用，9.絲帶： 未使用，10.針頭： 未使用，11.Vessel loop： 未使用，12.Boots： 未使用，13.Jelco： 未使用，14.棉條： 未使用，
    - 第一次：
        - 刷手護士 ： 周筱涵
        - 流動護士： 上次簽章: 張秀敏
        - 時間： 1320

，
    - 第二次：
        - 刷手護士 ： 周筱涵
        - 流動護士： 張秀敏 上次簽章: 張秀敏
        - 時間： 1325

，
    - 第三次：
        - 刷手護士 ： 周筱涵
        - 流動護士： 張秀敏 上次簽章: 張秀敏
        - 時間： 1330

備註

備註： DAVINCI 30度 SCOPE NO.1

- 記錄者： 上次簽章: 周筱涵

- 時間： 0745

填寫護理人員 張秀敏

正式摘要

	W52 / W52

doctorRank:B
